# Sleep Deprivation Induces Cognitive Impairment by Increasing Blood-Brain Barrier Permeability via CD44

**DOI:** 10.3389/fneur.2020.563916

**Published:** 2020-11-27

**Authors:** Jing Sun, Jusheng Wu, Fuzhou Hua, Yong Chen, Fenfang Zhan, Guohai Xu

**Affiliations:** ^1^Department of Anesthesiology, The Second Affiliated Hospital of Nanchang University, Nanchang, China; ^2^Department of Anesthesiology, Zhuji People's Hospital of Zhejiang Province, Shaoxing, China

**Keywords:** cognitive impairment, blood-brain barrier permeability, CD44, astrocytes, sleep deprivation

## Abstract

Sleep deprivation occurs frequently in older adults, which can result in delirium and cognitive impairment. CD44 is a key molecular in blood-brain barrier (BBB) regulation. However, whether CD44 participates in the role of sleep deprivation in cognitive impairment remains unclear. In this study, the effect of sleep deprivation on cognitive ability, tissue inflammation, BBB permeability, and astrocyte activity were evaluated *in vivo*. The differentially expressed genes (DEGs) were identified by RNA sequencing. A CD44 overexpression in the BBB model was performed *in vitro* to assess the effect and mechanisms of CD44. Sleep deprivation impaired the learning and memory ability and increased the levels of inflammatory cytokines, along with increased BBB permeability and activated astrocytes in hippocampus tissue. RNA sequencing of the hippocampus tissue revealed that 329 genes were upregulated in sleep deprivation-induced mice compared to control mice, and 147 genes were downregulated. GO and pathways showed that DEGs were mainly involved in BBB permeability and astrocyte activation, including nervous system development, neuron development, and brain development, and neuroactive ligand-receptor interaction. Moreover, the PCR analysis revealed that CD44 was dramatically increased in mice with sleep deprivation induction. The overexpression of CD44 in astrocytes promoted BBB permeability *in vitro* and induced the expression of the downstream gene NANOG. Our results indicate that sleep deprivation upregulated CD44 expression in hippocampus tissue, and increased BBB permeability, resulting in cognitive impairment.

## Introduction

Cognitive impairment is a characteristic seen in elderly patients with various diseases, such as Parkinson's disease, Alzheimer's disease, cardiovascular disease, and cancer (1–3), and is an important healthcare problem that is forecasted to worsen in the near future. Adequate cognition is crucial for understanding important facts, but the prevalence of impaired cognition increases sharply after the age of 65 and in people with a more advanced form of cognitive impairment (4). Hughes et al. found a significant association between sleep disturbance and cognitive dysfunction (5). Sleep deprivation contributes to cognitive decline and the development of neurodegenerative diseases (6, 7). However, the mechanisms by which sleep deprivation impacts cognitive impairment is still not clear.

The blood-brain barrier (BBB) is a dynamic multicellular interface between the central nervous system (CNS) and the blood circulatory system, which regulates material exchange between the circulation and the brain parenchyma, and maintains the homeostasis of the CNS (8). The BBB is mainly composed of brain microvascular endothelial cells, astrocytes, glial endfeet, pericytes, and the basement membrane (BM) (9). Brain microvascular endothelial cells interconnected with tight junctions, form the BBB's primary barrier (10, 11). Disruption of the BBB is a leading factor associated with the development of postoperative cognitive dysfunction (POCD). Zhu et al. found that peripheral immune cells participated in the inflammatory reaction within the hippocampus, following the administration of anesthesia via inhalation and the destruction of the BBB (12). Increasing BBB permeability leads to harmful elements, such as pro-inflammatory factors, reactive oxygen species, and neurotoxins, which infiltrate into the brain and trigger neural injury (13). A study by Ni et al. showed that surgery destroyed the BBB and led to cognitive dysfunction in aged rats (14).

Astrocytes are an important component of the BBB as well as the tripartite synapse neural network and promote the bidirectional communication of various materials under physiological conditions (15). Vitamin D3 improves disruption to the BBB by upregulating endogenous osteopontin (OPN) in the astrocytes and subsequent CD44 splicing (16), the elevated CD44 ligand hyaluronic acid promotes BBB permeability (17). Astrocytes play an important role in cognitive function. Price et al. addressed the potential role of astrocytes in the pathophysiology of vascular contributions to cognitive impairment and dementia (VCID) (18), and astrocytes act as new targets to improve cognitive functions (19). Interestingly, the previous study showed that sleep and wake time strongly affected the gene expression and ultrastructure of astrocytes in the mouse brain (20). These studies suggested that sleep deprivation, astrocytes, and the BBB are associated with cognitive impairment. However, it remains unknown whether sleep deprivation can induce cognitive impairment through regulating BBB permeability.

In this paper, we aimed to study the mechanisms of sleep deprivation on cognitive ability. Sleep deprivation models in aged mice were performed to explore the effect of sleep deprivation on cognitive ability, tissue inflammation, BBB permeability, and astrocyte activity. An RNA sequencing technique was used to identify the vital genes involved in BBB permeability. Moreover, we explored the roles of vital genes in BBB permeability *in vitro* by overexpressing the vital genes in astrocytes.

## Materials and Methods

### Animals

The adult male C57 BL/6J mice (18 months, *n* = 18) used in this study came from Yingbio Technology (Shanghai, China), and were housed in a temperature and humidity-controlled environment (12 h light: 12 h dark cycle) with free access to food and water. The animal experiment in this study was approved via the animal care and ethical committee of the Second Affiliated Hospital of Nanchang University. The use of the mice was carried out following the guidelines of the China legislation on the ethical use and care of laboratory animals.

### Establishment of the Sleep Deprivation Model

The sleep deprivation model was induced using an inverted flowerpot in a water tank (21). Briefly, sleep deprived (SD, *n* = 9) mice were placed on an inverted flowerpot (platform of 1.1 cm diameter) surrounded by a water-filled chamber of Plexiglas. The water reached a level of 2 cm below the base of the platform and was maintained at a temperature of 30 ± 1°C. Food and water were freely available to the animals. Control mice (Con, *n* = 9) were raised in normal cultivation. The time of sleep deprivation was 72 h (22, 23).

The learning and memory function in each group (*n* = 3) were examined using a Morris water maze assay as in a previous report (24, 25). The Morris water maze system used in our study consisted of a water maze pool, camera system, and an animal behavior trajectory analysis system. Briefly, the mice participated in daily trials four times a day for 5 consecutive days (time gap of each trial: 30 min). The escape latency time required for mice from entering the water to standing on the platform was recorded. The final result of 1 day was the average across the four trials. The escape latency was applied to determine the space learning and memory ability. The action trajectory diagram of the last experiment was recorded.

### Evans Blue Extravasation

BBB permeability was assessed by Evans Blue (EB) extravasation in three mice per group (26–29). EB dye (2% in saline, 4 ml/kg) was injected into the tail vein. After 3 h, mice were deeply anesthetized with 2 ml pentobarbital sodium, and perfused with 50 ml physiological saline through the left ventricle to remove the intravascular dye until a colorless perfusion fluid was observed from the right atrium. Mice were executed by cervical dislocation and the whole brain was removed quickly. The whole brain was immersed in 1 ml of formamide at 37°C for 2 h, then centrifuged at 20,000 × g for 20 min. The absorbance at 632 nm of the whole brain was measured using a spectrophotometer. EB concentrations were calculated against a standard curve using the Oringen7.0 software, and the obtained results were expressed as μg/g brain tissue.

### Hippocampus Tissues

Mice were deeply anesthetized and executed by cervical dislocation. The hippocampus tissues were removed and dissected and frozen in liquid nitrogen immediately, and stored at −80°C until they were needed for later analysis.

### Reverse Transcription-Quantitative Polymerase Chain Reaction

RNA was extracted from the mouse hippocampus using Trizol (Invitrogen) following the manufacturer's protocol. The quantity and quality of RNA were analyzed by microspectrophotometer (TGem, TIANGEN). RNA (1 μg/sample) was reverse transcribed to cDNA with the Reverse Transcription Kit (Thermo). A reverse transcription-quantitative polymerase chain reaction (qRT-PCR) was performed with the SYBR Green MasterMix (Roche) by Applied Biosystems Inc (ABI QuantStudio 6, Flex). The PCR conditions were 10 min at 95°C, 45 cycles of 15 s denaturation at 95°C, 60 s annealing at 60°C, and fluorescence collection from 60 to 99°C. All PCR primer sequences (Table 1) were synthesized by Shanghai Sangon Biological (Shanghai, China). GAPDH was used as an internal control. Data were obtained as cycle threshold (Ct) values, and the relative expression of mRNA was determined by using the 2^−ΔΔ*Ct*^ method.

**Table 1:** Primer sequences of mRNA for qRT-PCR.

**Primer**	**Sequences (5^**′**^ to 3^**′**^)**	**Expected product**	**Tm/^**°**^C**
		**length/bp**	
GAPDH-F	CAAAATGGTGAAGGTCGGTGT	118	60
GAPDH-R	GAGGTCAATGAAGGGGTCGTT		
VE-Cadherin-F	TCGTGGTGGAAACACAAGATG	113	60
VE-Cadherin-R	TGTGGATTGAGTAAAGACGGGG		
ZO-1-F	GGGGATGTTGTCTTGAAGATAAATG	100	60
ZO-1-R	CCATTTTTAACTTGCCTTTAGACCT		
spp1-F	TCCAATCGTCCCTACAGTCG	228	59
spp1-R	CCCTTTCCGTTGTTGTCCTG		
Met-F	AGAACGCTTGGCATGTGATC	180	59
Met-R	GTTTCTGCCGTGAAGTTGGG		
Sema3a -F	TTCTCTGGCCGCACAATAC	155	59
Sema3a -R	TGGCACATTGTTCTTTCCGT		
Epha7-F	TCTAAAGAGCTGCGACCCAA	180	59
Epha7-R	TGTTGTGCTTTCGAGTCCAG		
CD44-F	GCGACTAGATCCCTCCGTTT		
CD44-R	GGAGATACTGTAGCGGCCAT		
NANOG-F	AAAGGATGAAGTGCAAGCGG	115	60
NANOG-R	TGGGGATAGCTGCAATGGAT		
RHOA-F	CGGGAGTTGGACTAGGCAAG	114	60
RHOA-R	ATCCACCCAAACCCTCACTG		
Cdc42-F	CACCCAACCATGCGTCCC	111	60
Cdc42-R	CTTGTCCTCAGCTTCTCCGC		

### Western Blotting

Protein in the mouse hippocampus was extracted with an ice-cold RIPA buffer containing a cocktail of protease inhibitors. Protein concentration was measured by a BCA assay kit. Protein samples were separated in 10–15% SDS-PAGE gels and transferred to polyvinylidene fluoride membranes (Millipore). Membranes were blocked with 5% milk in TBS with 0.1% (v/v) Tween-20 (TBST) for 2 h at room temperature and incubated overnight at 4°C with primary antibodies. The primary antibodies applied are as follows: anti-vascular endothelial (VE)-Cadherin (ab205336, 1:1,000, Abcam), anti-zonula occludens-1 (ZO-1, Ab61357, 1:1,000, Abcam), anti-glial fibrillary acidic protein (GFAP, Cat.#80788, 1:1,000, Cell Signaling Technology), claudin-5 (ab131259, 1:2,000, Abcam), NANOG (sc-134218, 1:1,0000, Aanta Cruz), and GAPDH (60004-1-Ig, 1:1,000, Proteintech). The membranes were then washed three times with TBST for 10 min each and incubated with a secondary antibody (goat anti-mouse IgG-HRP antibody, ab205719, 1:20,000, Abcam; goat anti-rabbit IgG-HRP antibody, ab6721, 1:20,000, Abcam). Again after three washes with TBST, membranes were subjected to ECL chemiluminescence reagent (Thermo). Images were obtained using a ChemiDoc Gel Imaging system (Thermo), and the optical density was quantified with the Image J (v1.8.0) analytical software. Experiments were performed in triplicate.

### ELISA

The mouse hippocampus was collected, and the protein extraction was performed as in the previous method. IL-6 (EK0411, Boster Biological Technology Co. Ltd), IL-1β (EK0394, Boster Biological Technology Co. Ltd), and tumor necrosis factor (TNF)-α (EK0527, Boster Biological Technology Co. Ltd) ELISA kits were used to measure the levels of IL-6, IL-1β, and TNF-α in the mouse hippocampus tissue following the instructions from the manufacturer.

### Immunohistochemistry

GFAP expression in the mouse hippocampus was measured using immunohistochemistry (IHC). The mouse hippocampus was perfused through the heart with 4% paraformaldehyde. Paraffin sections were prepared and deparaffinized through dimethyl benzene and ethanol, and antigens were retrieved by incubation in a citric acid buffer (pH 6.0) at 100°C for 20 min. The sections were incubated with 3% H_2_O_2_ for 10 min to block any endogenous peroxidase activity and then with 5% BSA for 35 min to block non-specific binding. Primary anti-GFAP antibodies (ab68428, 1:400, Abcam) were then added and incubated at 4°C for overnight. After washing three times with PBS, the sections were incubated with the goat anti-rabbit IgG antibody (ab6721, 1:8,000, Abcam) for 35 min at 37°C. The sections were stained with the DAB Horseradish Peroxidase Color Development Kit and then dyed with hematoxylin. Finally, the sections were imaged using a microscope.

### Library Preparation and Sequencing

Total RNA was extracted and evaluated using the previous method. The sequencing library was generated by using the mRNA-seq Library Prep Kit for Illumina (Vazyme Biotech, China), following the manufacturer's protocols. In brief, mRNA (1–4 μg) was separated by Capture Beads, then the isolation mRNA was fragmented with the Frag/Prime Buffer. The cDNA was synthesized using the first Strand Mix, and the second Strand/End Mix. The cDNA was connected with adapters, sorted by fragment, and enriched with PCR. The sequencing of libraries was performed by an Illumina HiSeq 2500 platform (USA).

### Bioinformatics Analysis

The raw data derived from RNA-seq was analyzed by using Fast-QC (http://www.bioinformatics.babraham.ac.uk/projects/fastqc/) to filter away the adaptor sequences and low-quality sequences, and the clean reads were mapped to the mouse genome with HISAT2. The expression level of each gene was quantified as fragments per kilobase of exon per million mapped fragments (FPKM) and counts, and the DESeq2 algorithm was applied to filter the different expression genes (DEGs). The raw *p*-value was adjusted to the false discovery rate (FDR), and genes with FDR <0.05, fold change (log_2_ FC >1) were considered to be significantly differentially expressed. Gene ontology (GO, http://www.geneontology.org/) and Kyoto encyclopedia of genes and genomes (KEGG, http://www.genome.jp/kegg/) analyses were performed to evaluate the biological function of the DEGs.

### Cell Culture and CD44 Overexpression

The mouse astrocytes CP-M157 were purchased from the Procell Life Science & Technology Co., Ltd. (Wuhan, China) and cultured in ACM-57 (Shanghai Zhong Qiao Xin Zhou Biotechnology Co., Ltd) supplemented with 10% Fetal Bovine Serum (FBS, 10099-141, GIBCO), and 1% penicillin-streptomycin (Cat.#0503, ScienCell) at 37°C in a humidified 5% CO_2_ atmosphere. When the cells seeded in 6-well plates were grown to 80–90% confluency, CD44 was transfected into the cells with pcDNA3.1-CD44 using Lipofectamine 2000 (Invitrogen), and the control cells were transfected with pcDNA3.1. After 24 h, the cells in one insert of the 6-well plates were harvested, qRT-PCR and Western blot were used to confirm the overexpression efficiency.

### Establishment of the BBB Model *in vitro* and Measurement of BBB Permeability

The mouse brain microvascular endothelial cells (BMECs) bEnd.3 (Catalog#1001, ScienCell) were cultured in an endothelial cell medium with an endothelial cell growth supplement (ECGS, Cat.#1052, ScienCell), 10% FBS, 1% fetal bovine serum, and 1% penicillin-streptomycin. To set up a BBB model *in vitro*, astrocytes were seeded on the bottom of the collagen-coated Transwell chamber (Corning #3413) with 5.0 × 10^5^ cells/chamber, the Transwell chamber was inverted and the cells were cultured for 4 h, the chamber was then flipped and placed into six-well plate. After 7 days of culture, 2.5 × 10^5^ cells/cm^2^ BMECs were added to the top of the Transwell chamber. The astrocyte culture medium (1 ml) was added into the vascular endothelial cells for co-culture. The barrier integrity of BMECs was evaluated using transendothelial electrical resistance (TEER) values and the permeability of sodium fluorescein (NaF) (30). After incubation for 1 week, the culture medium was changed to a serum-free medium, and 100 μg/ml of NaF and donor pool was added. After 2 h incubation at 37°C, 100 μl of the culture medium was removed to determine that the concentrations of the fluorescein in samples were detected by a fluorescence spectrophotometer. The BBB permeability was calculated as the reference. The integrity of the monolayers was assessed by TEER with a Millicell-Electrical Resistance System (USA). The expression of tight junction proteins in BMECs were detected using Western bolt. One insert of 6-well plates were used for each experiment.

### Statistical Analysis

Statistical analysis was performed using the SPSS 24.0 software. Data were expressed as the mean ± SD, and a Student's *T*-test was used to determine statistical differences. A *p* < 0.05 was considered as statistically significant.

## Results

### Sleep Deprivation-Induced Cognitive Impairment

**Figure 1:**
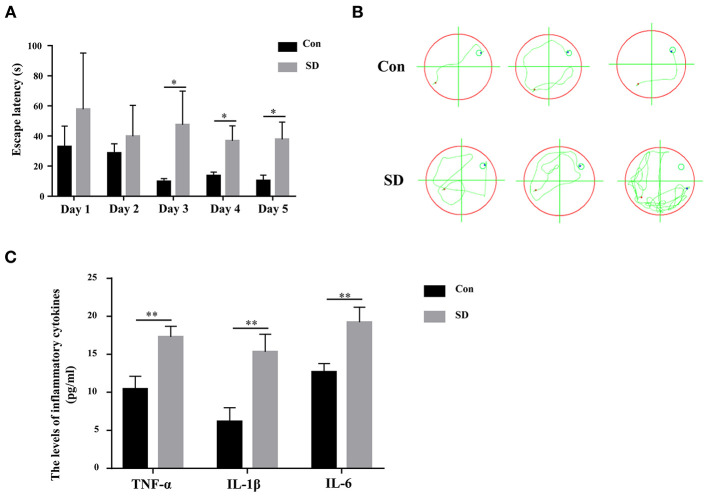
Sleep deprivation induced cognitive impairment. **(A)** The escape latency during 5 days of training and **(B)** The trajectory of the last experiment in a Morris water maze assay was used to measure the learning and memory ability. **(C)** The expression of inflammatory cytokines (IL-6, IL-1β, TNF-α) was detected by an ELISA assay kit. Data are mean ± SD of at least three duplicate experiments. *p < 0.05; **p < 0.01 (Student's *t*-test).

To investigate the effect of sleep deprivation on the cognitive ability of mice, we constructed a sleep deprivation mice model. A Morris water maze assay was subsequently used to assess the learning and memory ability of aged mice with sleep deprivation. During the 5 days of the experiment (four trials per day, with time intervals of 30 min), the escape latency of model mice was higher than that of the control mice until the third day when the escape latency had a significant difference (Figure 1A). Moreover, in the last experiment, the trajectory of mice with sleep deprivation induction was disordered compare to that in the control mice, suggesting the cognitive of the model mice was impaired (Figure 1B). These results indicated that the sleep deprivation model was successfully constructed, and sleep deprivation triggered the cognitive impairment of elderly mice. Additionally, the inflammatory cytokines (IL-6, IL-1β, TNF-α) in the hippocampus tissue from model mice were significantly increased as compared to the control mice (Figure 1C).

### BBB Permeability Was Increased in Sleep Deprived Mice

**Figure 2:**
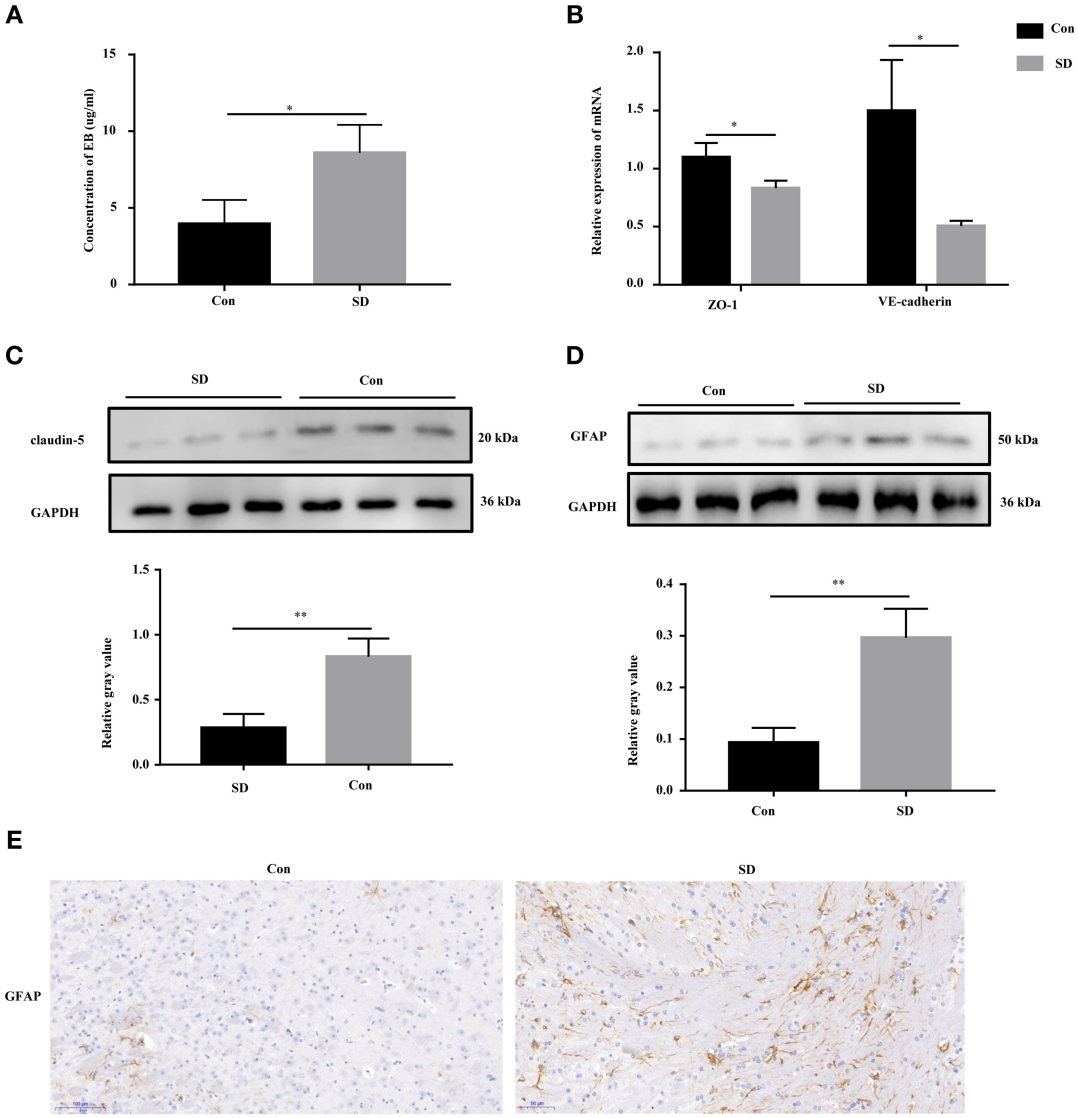
BBB permeability was increased in sleep deprivation mice. **(A)** The level of EB was performed to assess BBB permeability in mice. **(B)** The relative mRNA levels of VE-Cadherin and ZO-1 were measured by qRT-PCR. The levels were expressed as the value of 2^−ΔΔ*Ct*^. **(C)** The expression of claudin-5 in the hippocampus tissues of mice was detected by Western blot. **(D,E)** The expression of GFAP in the hippocampus tissues of mice was detected by Western blot and immunohistochemistry (400x). Data are mean ± SD of at least three duplicate experiments. *p < 0.05; **p < 0.01 (Student's *t*-test).

Previous studies have shown that cognitive impairment is strongly associated with BBB permeability (31). Thus, we first identified the effect of sleep deprivation on BBB permeability of the model mice. The concentration of EB in sleep deprived mice was significantly increased in comparison with the control mice, indicating that BBB permeability was elevated (Figure 2A). The maintenance of BBB integrity is dependent on endothelial tight and adherence junctions, which can be measured via detecting the expression of their biomarkers, including VE-Cadherin, ZO-1, and claudin-5 (32, 33). In sleep deprived mice, the mRNA and protein levels of VE-Cadherin, ZO-1, and claudin-5 were significantly reduced compared to the control mice (Figures 2B,C). Subsequently, in the hippocampus region of the model mice, we tested whether astrocytes were activated by assessing GFAP (a marker of astrocytes) expression to identification. Western blotting and immunohistochemistry results showed that GFAP expression was remarkably increased in the hippocampus tissues of deprivation mice compared to that of control mice (Figures 2D,E).

### Overview of Sequencing Data and DEGs

**Figure 3:**
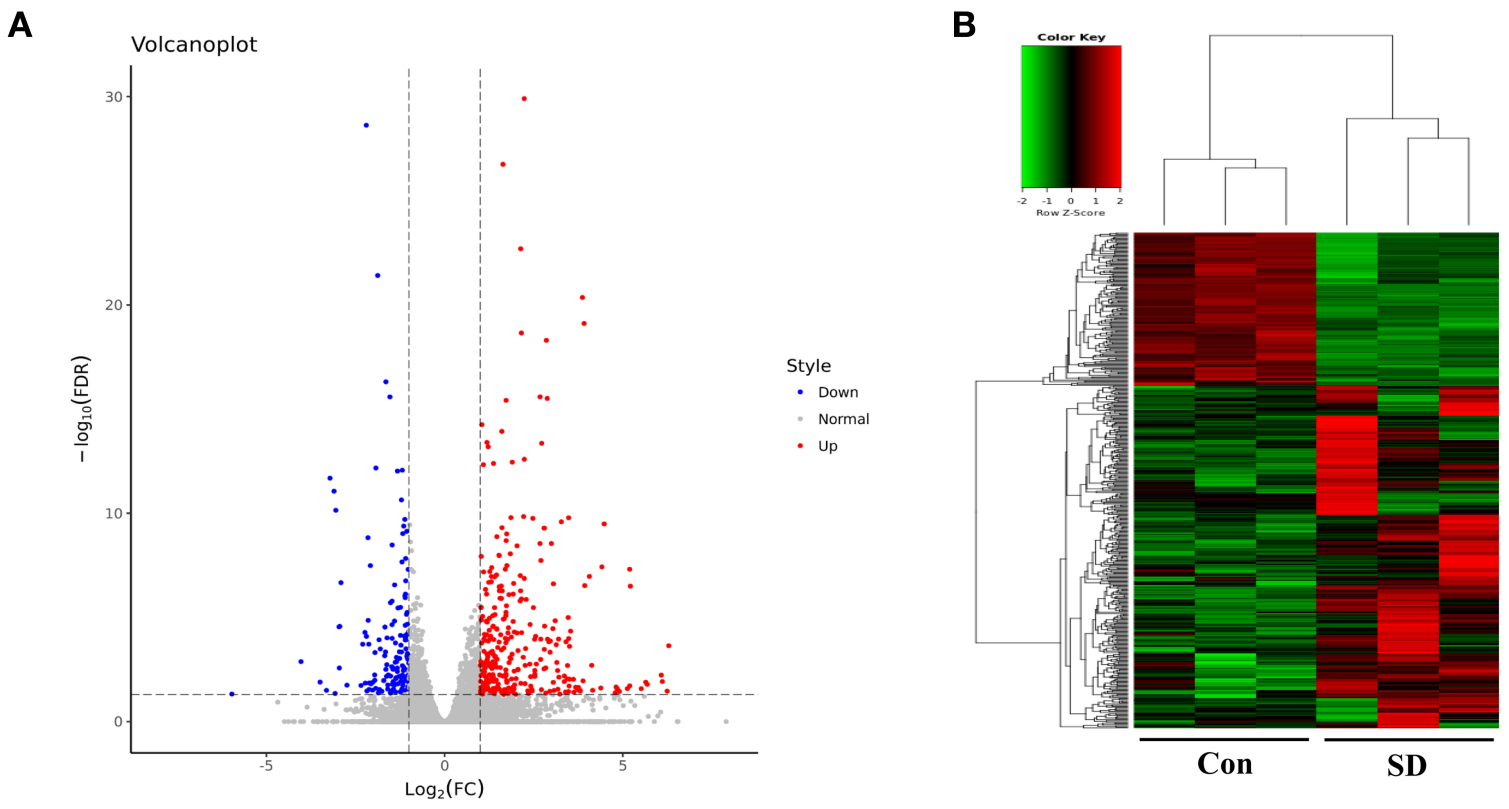
Overview of sequencing data and DEGs. **(A)** A volcano plot was used to analyze the DEGs in sleep deprived mice and control mice. The x-axis denotes log_2_ (fold change) of genes between SD mice and control mice, and y-axis denotes the FDR value (–log_10_ transformed) of genes. Upregulated genes are shown as red color and downregulated as blue color. **(B)** The expression levels of DEGs in SD mice and control mice were clustered in the heat map. The high expression to low expression was shown as red to green color.

To study the effect of sleep deprivation-related-mRNA on BBB permeability, we performed RNA sequencing. Followed by raw read filtering, approximately 44, 36, and 33 million clean reads were, respectively, generated in three model mice samples (SD 1, SD 2, and SD 3), and 34, 36, and 35 million high-quality clean reads were, respectively, obtained in three control mice samples (Con 1, Con 2, and Con 3). A total of 96–97% of clean reads in both groups were fully mapped to the reference genome (Table 2). Of note, a total 329 DEGs (upregulation) and 147 DEGs (downregulation) were found in the two groups (Figure 3A). The expression difference of DEGs were visualized through the heat map, in which the DEGs were clearly self-isolated into SD and Con clusters (Figure 3B).

**Table 2:** Summary of the sequencing reads alignment to the reference genome.

	**All reads**	**Clean reads**	**Mapped reads**	**Mapped rate (%)**
Con 1	37045066	33871804	32713328	96.57982197
Con 2	37901468	35710496	34525669	96.68213233
Con 3	36950246	34746666	33721617	97.04993567
SD 1	46437262	43898458	42648308	97.15217787
SD 2	38291828	36175114	35184241	97.26089875
SD 3	34490242	32552430	31698308	97.37616516

### Functional and Pathway Analysis of DEGs

**Figure 4:**
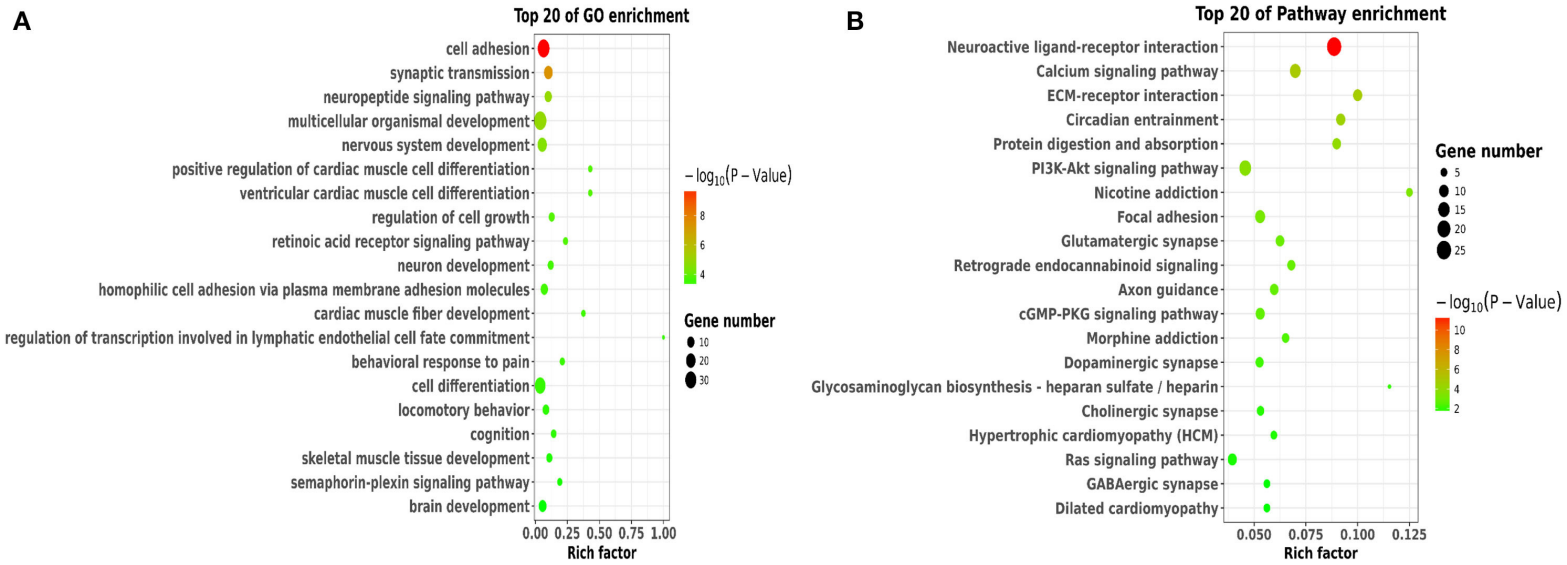
Functional and pathway analysis of DEGs. **(A)** The enrichment analysis of GO terms based on DEGs in sleep deprived mice relative to control mice. **(B)** Top 20 significant KEGG terms based on DEGs in sleep deprived mice relative to control mice.

To better understand the potential roles of these DEGs, we performed GO and KEGG enrichment analysis. Notably, GO analysis (Figure 4A) showed that DEGs were mainly enriched in cell adhesion, synaptic transmission, nervous system development, neuron development, and brain development, which were involved in BBB permeability (34–38). The pathway analysis of DEGs using KEGG analysis are shown in Figure 4B. We found that various signaling pathways were implicated in BBB permeability and inflammatory response, including the neuroactive ligand-receptor interaction (39), calcium signaling pathway (40), circadian entrainment (41, 42), and PI3K-Akt signaling pathway (43).

### The Expression of Candidate Genes Verified by qRT-PCR

**Figure 5:**
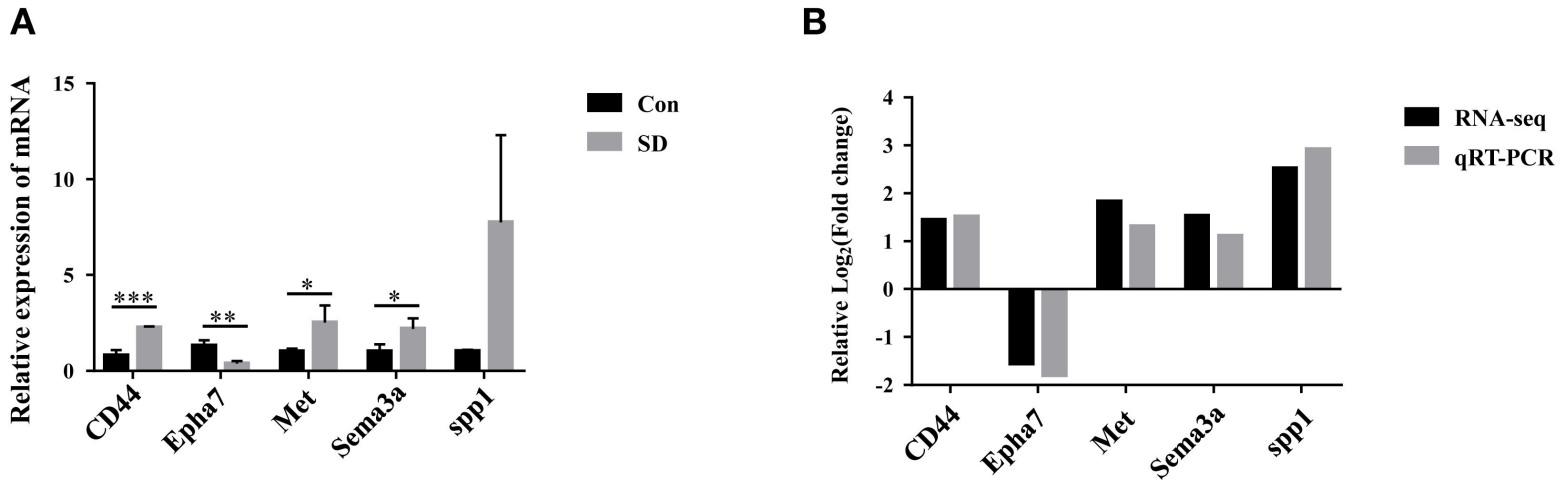
The expression of candidate genes verified by qRT-PCR. **(A)** The relative expression of candidate genes in hippocampus tissues was measured by qRT-PCR. The levels were expressed as the value of 2^−ΔΔ*Ct*^. **(B)** Comparison of RNA-seq and qRT-PCR results. Data are mean ± SD of three duplicate experiments. *p < 0.05; **p < 0.01; ***p < 0.001 (Student's *t*-test).

To identify the key DEG regulated by sleep deprivation, the five genes (CD44, Met, Sema3a, spp1, Epha7) were selected according to their high fold change value and high abundance, associated with BBB and astrocytes activation. The qRT-PCR results showed that the expression of CD44, Met, Sema3a, and spp1 were upregulated, and that of Epha7 was significantly downregulated in model mice compared to the control group (Figure 5A). The RT-qPCR validation results were generally in accordance with the sequencing results (Figure 5B). Importantly, we selected CD44 as the research object because CD44 had a significant statistical difference and a relative higher fold change value than other genes, and is implicated in the BBB (42, 44).

### Overexpression of CD44 Facilitated BBB Permeability of Astrocytes *in vitro*

**Figure 6:**
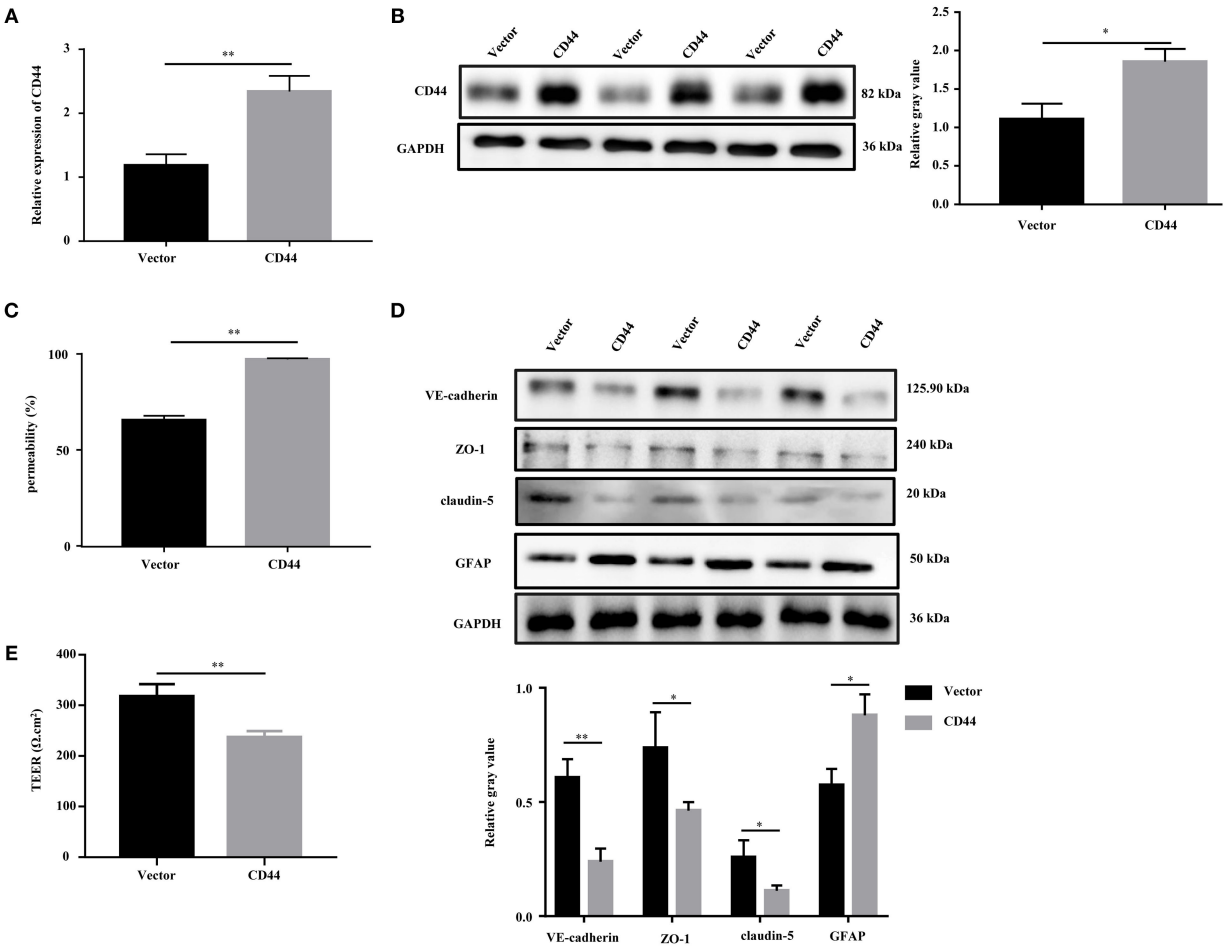
Overexpression of CD44 facilitated the BBB permeability of astrocytes *in vitro*. **(A,B)** The relative mRNA and protein expression of CD44 in mouse astrocytes were measured by qRT-PCR and Western blot, respectively. The mRNA level were expressed as the value of 2^−ΔΔ*Ct*^. **(C)** NaF was employed to evaluate BBB permeability. **(D)** The protein levels of VE-Cadherin, ZO-1, claudin-5, and GFAP were measured by Western blot. **(E)** The integrity of the BBB was assessed by TEER. Data are mean ± SD of three duplicate experiments. *p < 0.05; **p < 0.01 (Student's *t*-test).

To evaluate the effect of CD44 on BBB permeability, we first transfected the CD44 overexpression vector into mouse astrocytes. The qRT-PCR and Western blot analysis revealed that the expression of CD44 was increased compared to the control vector (Figures 6A,B). Furthermore, the BBB model *in vitro* was set up by the co-incubation of mouse astrocytes with mouse brain microvascular endothelial cells bEnd.3. After CD44 overexpression, BBB permeability in the BBB model was significantly increased compared to the control vector (Figure 6C). With the overexpression of CD44 in mouse astrocytes, the expression levels of VE-cadherin, ZO-1, and claudin-5 in bEnd.3 of the BBB model were decreased compared to the control model, and the expression of GFAP was induced (Figure 6D). In addition, the TEER value of the BBB model with CD44 overexpression was decreased, indicating that the breakdown of BBB integrity (Figure 6E). Collectively, the above findings suggested that BBB permeability might be affected via upregulating CD44 in astrocytes.

### Overexpression of CD44 in Astrocytes Promoted the Expression of Downstream Target Gene NANOG

**Figure 7:**
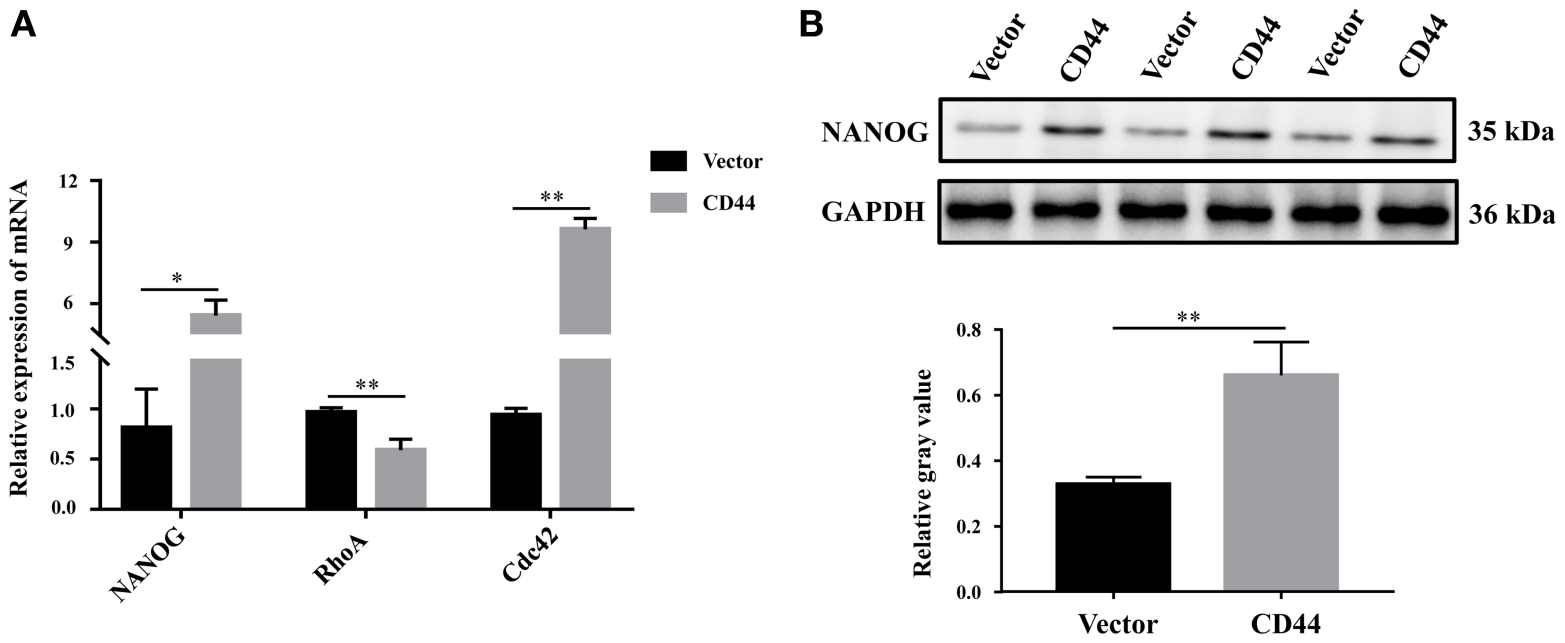
Overexpression of CD44 in astrocytes promoted the expression of the downstream target gene NANOG. **(A)** The relative mRNA levels of RhoA, NANOG, and Cdc42 were examined with qRT-PCR. **(B)** The protein level of NANOG was confirmed using Western blot. Data are mean ± SD of three duplicate experiments. *p < 0.05; **p < 0.01 (Student's *t*-test).

To better understand the molecular regulatory mechanism that mediates BBB permeability, we sought the downstream genes of CD44. Three downstream genes (RhoA, NANOG, Cdc42), which were related to BBB permeability (45–48), were selected for further study. The protein levels of NANOG and Cdc42 were increased in astrocytes with CD44 overexpression, and the expression of RHOA was decreased (Figure 7A). Literature inquiries found that RhoA mediated the expression of the inflammatory gene in astrocytes and increased BBB permeability (45, 49). The assembly of tight junction proteins was triggered by the activation of Cdc42, resulting in BBB tightness (50). The NVs-mediated miR-34a delivery system decreased CD44 expression, and reduced the expression of the NANOG genes, indicating the expression of the NANOG gene changed with CD44 (51). Notably, only the changes to NANOG in astrocytes with CD44 overexpression were consistent with the related literature. Therefore, we performed Western blotting analysis to validate the NANOG expression and found that the NANOG expression was significantly decreased in astrocytes with CD44 overexpression (Figure 7B).

## Discussion

As a common feature of various diseases, cognitive impairment impacts the health of elderly patients (52). Previous studies showed that BBB permeability has an essential effect on cognitive impairment-related diseases (53), but the function and mechanism of BBB permeability in cognitive impairment caused by sleep deprivation is unclear. Our study revealed that the learning and memory ability of SD mice were impaired by sleep deprivation, concomitant with the increasing BBB permeability. RNA sequencing and PCR validation identified that CD44 was increased in sleep deprived mice compared to the control mice. Moreover, the overexpression of CD44 in astrocytes facilitated BBB permeability *in vitro* and induced the expression of the downstream target gene NANOG.

Sleep and its disorders are known to influence the BBB. Sleep loss impairs the function of the BBB by disrupting the interactions of pericytes and brain endothelial cells (54). Sleep participates in the neural biological regulation by facilitating the elimination of metabolites along the BBB (55). As in severe stress, sleep deprivation impairs BBB function and induces brain pathology (21). Similarly, our study also showed that BBB permeability was significantly increased in mice with sleep deprivation induction, suggesting the vital effect of sleep deprivation on the BBB.

CD44, a receptor for the extracellular matrix component hyaluronan, is a widely expressed cell adhesion molecule (56). CD44 plays a vital role in different events involved in the inflammatory process and in several neurological disorders. By elevating CD44 expression, hyaluronan blunted the barrier integrity of brain microvascular endothelial cells (42). The increase of BBB permeability is associated with elevated CD44 ligand hyaluronic acid levels (17), suggesting that CD44 can promote BBB permeability. Astrocytes with CD44 induction improved the tissue repair following CNS injury, and CD44 was highly expressed in astrocytes of the injured areas of the CNS (57, 58). The astrocyte inflammatory responses were attenuated by glycoprotein GPNMB via the CD44 receptor (59). Similarly, our study showed that the expression of CD44 was significantly upregulated in the sleep deprivation model, and the overexpression of CD44 in astrocytes induced BBB permeability.

RhoA, NANOG, and Cdc42 were selected to understand the downstream regulatory mechanism. Here, we noted these genes may play important roles in BBB permeability. The activation of RhoA in astrocytes has a unique effect in inducing the inflammatory responses of CNS disease progression (45), and Netrin-1 restored BBB integrity by inhibiting the RhoA signaling pathway (60). NANOG promotes the dedifferentiation of p53-deficient mouse astrocytes through changes to cell fate and transforming cell properties (47). NANOG induced the proliferation of astrocyte cells by interacting with CDK6 (61), and NANOG contributed to the mediation of the cellular transforming activity (62). In particular, NANOG was considered to initiate Sox2 transcription (63), and Sox2 increased the breakdown of the blood-brain barrier (64). Induction of Cdc42/Rho kinase in rat astrocytes regulated the cholesterol efflux (65). Notably, RhoA induced the expression levels of the inflammatory gene in astrocytes and facilitated BBB permeability (45, 49), Cdc42 activation mediated the assembly of tight junction proteins and caused BBB tightness (50), and the levels of the NANOG gene declined with CD44 expression (51). Our study showed the changing trend of RhoA and Cdc42 in CD44 overexpressed astrocytes, which facilitated BBB permeability *in vitro*, and were anticorrelated with literature reports. The changing trend of NANOG significantly decreased in CD44-overexpressed astrocytes, suggesting that CD44 might promote BBB permeability via NANOG.

In conclusion, this study revealed that sleep deprivation induced cognitive impairment and increase BBB permeability, along with activating astrocytes and inflammatory responses *in vitro*. Sequencing analysis identified that CD44 was markedly upregulated in cognitively impaired mice. Moreover, the overexpression of CD44 in astrocytes elevated BBB permeability *in vitro* and induced the expression of NANOG in astrocytes. Our findings provided a molecular basis for cognitive impairment related to sleep deprivation.

## Data Availability Statement

The raw data supporting the conclusions of this article will be made available by the authors, without undue reservation.

## Ethics Statement

The animal study was reviewed and approved by the Animal Care and Ethical Committee of Second Affiliated Hospital of Nanchang University.

## Author Contributions

GX and JS: guarantors of integrity of the entire study, study concepts, study design, and manuscript review. JS and FH: definition of intellectual content and manuscript preparation. JS and JW: literature research and manuscript editing. JS, JW, FH, YC, and FZ: experimental studies. JS, JW, and FZ: data acquisition. YC, FH, and FZ: data analysis. JW and FZ: statistical analysis. All authors read and approved the final manuscript.

## Conflict of Interest

The authors declare that the research was conducted in the absence of any commercial or financial relationships that could be construed as a potential conflict of interest.
